# Color aberration in malachite kingfishers: Insights from community science observations in Queen Elizabeth National Park, Uganda

**DOI:** 10.1002/ece3.11717

**Published:** 2024-07-07

**Authors:** Bethany H. Warner, Katherine C. B. Weiss, Maximilian L. Allen

**Affiliations:** ^1^ Animal Sciences, College of Agricultural, Consumer, and Environmental Science University of Illinois Champaign Illinois USA; ^2^ School of Life Sciences Arizona State University Tempe Arizona USA; ^3^ Illinois Natural History Survey, Prairie Research Institute University of Illinois Champaign Illinois USA

**Keywords:** *Corythornis cristatus*, hypopigmentation, kingfisher, leucism, participatory science, Queen Elizabeth National Park

## Abstract

Color aberrations in birds corresponds with important ecological functions, including thermoregulation and physiological impacts, camouflage and increased predation, and social interactions with conspecifics. Color aberrations in birds have been reported frequently in the scientific literature, but aberrations in many species remain undocumented or understudied. We investigated records of leucism in malachite kingfishers (*Corythornis cristatus*) from observations of community scientists on iNaturalist and eBird in Uganda. Leucistic kingfishers were only observed within the Queen Elizabeth National Park (QENP), Uganda. When considering all observations of malachite kingfishers that included photographs within the QENP, leucistic individuals accounted for 13.0% and 10.4% of total malachite kingfisher observations within the study area from iNaturalist and eBird, respectively. Leucistic observations were recorded from September 2015 through February 2017, making up 60.0% and 68.2% of observations of malachite kingfishers within the study area from iNaturalist and eBird during that time, respectively. The localized and short documentation period suggests observations represent a single individual, while the high observation rate likely corresponds with collection bias due to the novelty of the individual. Our findings help to better understand the ecological importance and potential consequences for color‐aberrant individuals, although color aberration did not appear to inhibit our subject's ability to find a mate. Our work also highlights how participatory science can promote the documentation of color‐aberrant individuals in wild populations, although it poses challenges when trying to estimate abundance.

## Graphical abstract

We investigated records of hypopigmentation in malachite kingfishers (Corythornis cristatus) from observations of community scientists on iNaturalist and eBird in Uganda. Hypopigmented individuals accounted for 13.0% and 10.4% of total malachite kingfisher observations within the study area from iNaturalist and eBird, respectively; but from September 2015 through February 2017, hypopigmented individuals made up 60.0% and 68.2% of observations. The localized and short documentation period suggests observations represent a single individual, while the high observation rate likely corresponds with collection bias due to the novelty of the individual.

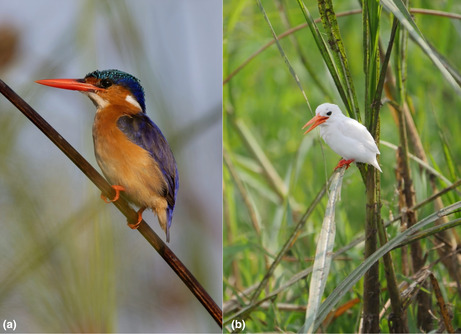

## 1 INTRODUCTION

Birds have some of the most unique colorations in nature, with these colorations attributed to the deposition of pigments (e.g., carotenoids and melanins; Galván & Solano, 2016). Melanins appear as either eumelanin (responsible for black, gray, and dark brown colors) or pheomelanin (responsible for red‐brown to buff colors), while carotenoids derived from the diet are responsible for yellow, orange, and red colorations (van Grouw, 2021). Blue, green, and violet coloration is attributed to spongy structures in the feather barbs and can vary depending on the scattering structure size and underlying pigments in the feather cortex (Finger, 1995). Abnormal coloration in birds is a consequence of inadequate pigment deposition resulting from melanin cell damage or mutations, including melanin defects in cell development, synthesis, or type produced (van Grouw, 2021). Melanin pigments are produced in melanocytes derived from melanoblasts through a complex array of signaling pathways that involve various genes and proteins (Arnheiter & Debbache, 2021; Wakamatsu & Ito, 2021). Hypopigmentation (i.e., lighter pigmentation) can be further divided into subcategories, such as albinism (the absence of tyrosinase in pigment cells, resulting in all‐white plumage, red eyes, and pink bill and feet; in avians it is the only form of color aberration that impacts eyesight; van Grouw, 2021), leucism (caused by a neural crest disorder leading to the absence of melanin cells in some or all areas of the body, resulting in all‐white or white spotting of plumage and in some cases pink feet and bill; van Grouw, 2021), and dilution (resulting from melanin being abnormally deposited in the cells, turning black/blue into silver/gray and red/yellow into buff/cream; the majority of dilution variants show normal coloration of feet and bill; van Grouw, 2021). Progressive graying is an alternative form of hypopigmentation that can present phenotypically similar to leucism but occurs after fledging unlike leucism which is congenital (Camacho et al., 2022).

Hypopigmentation can impact various aspects of avian ecology and behavior, because color adaptations affect sexual selection, social interactions, thermoregulation, photoprotection, and concealment (Delhey et al., 2023; Price‐Waldman & Stoddard, 2021). Some predators have been shown to focus on phenotypically distinct individuals within large groups, putting color aberrant individuals at a higher risk of predation (Rutz, 2012). Hypopigmentation can also impact social interactions and mate choice, introducing selection pressures that may contribute to population dynamics (Delhey et al., 2023). For example, in populations where birds use plumage signals to communicate, a hypopigmented individual may have difficulties establishing their social status (Davis, 2007). Melanin abnormalities resulting from hypopigmentation can also have physiological impacts on affected individuals. Melanic keratin in feathers is significantly harder than non‐melanic keratin, providing greater protection from feather‐degrading bacteria and a decreased chance of fractured feather barbs and barbules (Bonser, 1995; Galván & Solano, 2016). Hypopigmentation also changes protection against UV radiation, and alterations in pigmentation patterns can impact an individual's ability to regulate body temperature (Galván & Solano, 2016). Despite the extensive research on hypopigmentation's relation to individual fitness, there are still discrepancies in understanding its impact among different bird species. Although hypopigmentation is frequently associated with decreased individual fitness, some populations have shown evidence of potential selection for forms of hypopigmentation (i.e., leucism in Southern caracaras [*Caracara plancus*] in Patagonia; Edelaar et al., 2011). Further understanding is therefore needed on the ecological implications and potential costs of hypopigmentation in birds.

**FIGURE 1:**
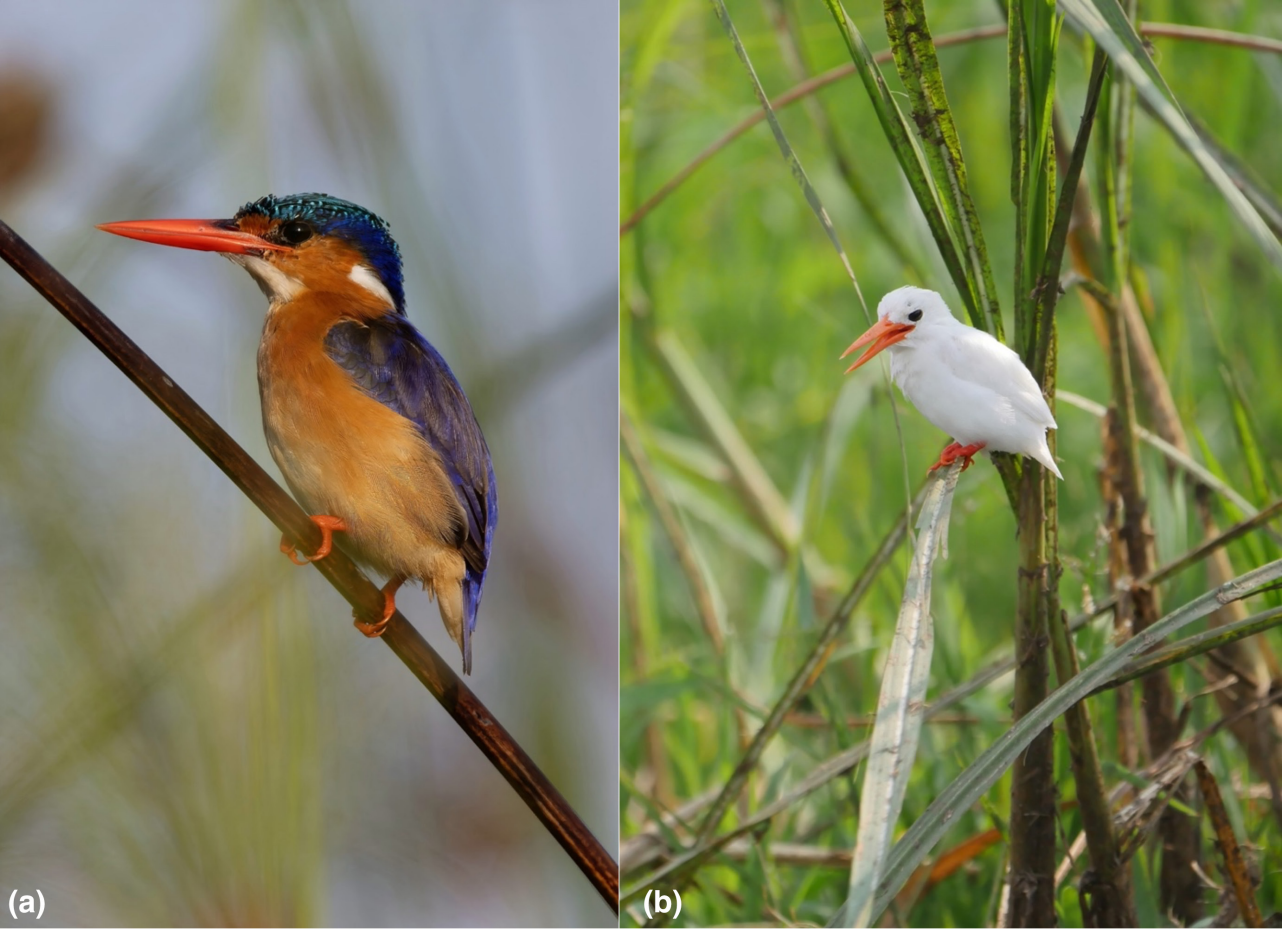
(a) Malachite kingfisher with normal coloration seen in Mabamba Swamp, Uganda, July 23, 2022. (b) Leucistic malachite kingfisher seen along the Kazinga Channel in the QENP, Uganda, July 29, 2016. Photos courtesy of Nik Borrow.

After noticing multiple observations of hypopigmented malachite kingfishers (*Corythornis cristatus*) in Uganda on iNaturalist, we chose to examine their occurrence and distribution. Malachite kingfishers have distinctive plumage coloration with a contrasting rusty orange underside, royal blue upperside, and a turquoise crown (Figure 1). The unique coloration of malachite kingfishers makes color‐aberrant individuals an interesting, albeit understudied, area of research. Although little is known about color aberrations in malachite kingfishers in Africa, studies have been published on color aberrations in other kingfisher species within India (Adhikary & Mondal, 2019; Das, 2023; Rathore & Saxena, 2022; Srinivasulu, 2004). We searched iNaturalist (www.inaturalist.com) and eBird (https://ebird.org) for observations of malachite kingfishers throughout their distribution and noted the location and occurrence of which observations were hypopigmented (all of which were within the Queen Elizabeth National Park (QENP)). We also performed a literature search of hypopigmented kingfishers to identify if prior observations have been documented within the literature.

## 2 METHODS

### 2.1 Study area

**FIGURE 2:**
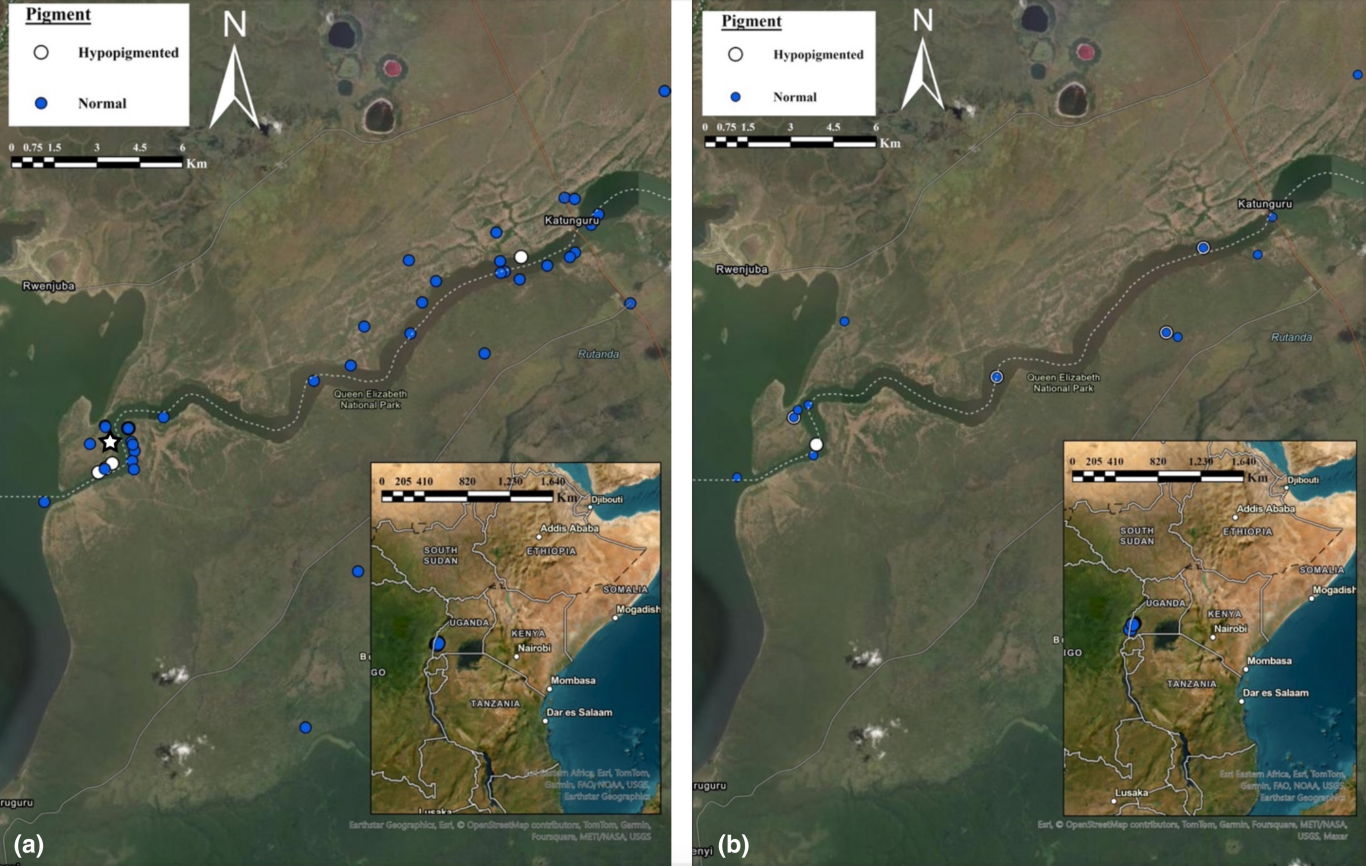
iNaturalist malachite kingfisher observations along the Kazinga Channel within the QENP, Uganda for both (a) iNaturalist and (b) eBird. Blue dots indicate observations of malachite kingfishers with normal coloration and white dots indicate hypopigmented (leucistic) malachite kingfisher observations. White star indicates reported iNaturalist location of leucistic malachite kingfisher pictured in Figure 1b.

Our study area was in the QENP in Western Uganda, with most observations concentrated along the Kazinga Channel (Figure 2). The park was founded in 1952 (Jones, 2013), is approximately 223,000 ha in size (Muhweezi, 2014), and ranges from 910 to 1350 m in elevation (Makanaga, 2023). The equatorial park has 12.1 h of daylight a day, with a consistent daily temperature averaging a high of 29°C during the day and a low of 17°C at night (Makanga, 2023). The park has two wet seasons (March–May, September–mid‐December) and two dry seasons (January–February, June–July) but rainfall varies within the park, with highs of 1250 mm in Maramagambo forest and 750 mm along Kazinga channel during the rainy season (Makanga, 2023). The park has an average high humidity of 84% and an average low humidity of 63% (Makanga, 2023). The vegetation in the park is largely associated with a savanna biome, but still has a wide variety with stretches of grasslands, woodlands, moist tropical forests, wetlands, and open bodies of water (Muhweezi, 2014).

### 2.2 Data collection

From December 9, 2023 to January 18, 2024 we performed a review of hypopigmented malachite kingfisher observations in Uganda on two social naturalist networks (iNaturalist and eBird) that provide biodiversity information from community scientists. All observations of hypopigmented kingfishers were located within the QENP. On iNaturalist, we only used research grade observations. Although a similar confirmation method is not available on eBird, we further narrowed our search to observations with images on both iNaturalist and eBird to confirm species identification and note if the individual in each observation was of normal coloration or noticeably hypopigmented.

We performed a literature search in Web of Science on January 17, 2024, to determine how frequently hypopigmented kingfishers have been documented in the scientific literature. We searched each genus name in Latin of kingfishers with the search terms albin*, leucis*, hypopigment* in English. We read each peer‐reviewed study found during our literature search and collated our findings. We also used snowball sampling, by reading studies referenced in the studies we read, to expand our search.

## 3 RESULTS

On iNaturalist, there were 46 malachite kingfisher observations within the QENP between September 2004 and December 2023, all localized around the Kazinga Channel (Figure 2a). Of these observations, six were hypopigmented (all from September 2015 through December 2016). Due to the small and localized number of occurrences, likely a single hypopigmented individual made up these observations, corresponding with 13.0% of observations overall, and 60.0% of observations when the hypopigmented individual was reported between September 2015 to December 2016.

On eBird, there were 144 malachite kingfisher observations within the QENP between March 2005 and December 2023, all localized around the Kazinga Channel (Figure 2b). Of these observations, 15 were hypopigmented (all from September 2015 through February 2017). The hypopigmented individual made up 10.4% of all observations, and 68.2% of observations from September 2015 to February 2017.

All observations of hypopigmented malachite kingfishers within the study area showed an individual with all white plumage, as well as normal coloration in the feet, beak, and eyes, suggesting the form of hypopigmentation is likely leucism. The only exceptions were one eBird and one iNaturalist observation that showed a dark feather on the left wing (https://macaulaylibrary.org/asset/206176941; https://www.inaturalist.org/observations/2399563).

Our systematic literature review found four previous reports of hypopigmented kingfishers from peer reviewed studies (Table 1). All four reports were from India and from four different species (Common kingfisher [*Alcedo atthis*]; Rathore & Saxena, 2022, Stork‐billed kingfisher [*Pelargopsis capensis*]; Das, 2023, Collared kingfisher [*Todiramphus chloris*]; Adhikary & Mondal, 2019, White‐throated kingfisher [*Halcyon smyrnensi*]; Srinivasulu, 2004). Three studies suggested leucism, and the fourth suggested albinism, but there is discourse on the accuracy of this claim outlined in Mahabal et al. 2016.

**TABLE 1:** Summary of peer reviewed reports on hypopigmentation in kingfisher species with the type of hypopigmentation reported in each article.

Study	Species	Location	Type of hypopigmentation	Description
Rathore & Saxena, 2022	*Alcedo atthis*	India	Leucism	Dark eyes, orange feet and beak, peppered dark feathers on wings, majority of plumage white
Das, 2023	*Pelargopsis capensis*	India	Leucism	Dark eyes, orange bill and feet, pale orange collar and underparts, white upperparts
Srinivasulu, 2004	*Halcyon smyrnensi*	India	Albinism	Considered albinism because plumage is white
Adhikary & Mondal, 2019	*Todiramphus chloris*	India	Leucism	Dark eyes, bill, and feet, all white plumage
This Study	*Corythornis cristatus*	Uganda	Leucism	Dark eyes, orange feet and beak, all white plumage

## 4 DISCUSSION

Although hypopigmentation has been documented in multiple bird species (van Grouw, 2021), we present the first, to our knowledge, documentation of color aberration among malachite kingfishers in the scientific literature. While our literature review found four previous instances of hypopigmented kingfisher species in peer‐reviewed literature in India (Adhikary & Mondal, 2019; Das, 2023; Rathore & Saxena, 2022; Srinivasulu, 2004), we also found a high incidence of observations of leucistic malachite kingfishers in the QENP. During the short time period when leucistic individuals were observed in the park (September 2015–February 2017), they accounted for 60.0% of malachite kingfisher observations on iNaturalist and 68.2% on eBird. It is likely that the QENP, as a popular tourist attraction, combined with the unique coloration of leucistic malachite kingfishers contributed to the disproportionately high observation rate. One iNaturalist participant reported seeing the leucistic malachite kingfisher posted to the social media platform Facebook in November 2015 and the individual has been highlighted in several African safari blogs, which may have encouraged other birders to travel to the QENP to catch a glimpse of this natural phenomenon. Previous studies have shown a collection bias for color‐aberrant birds that are easily detectable and so gain more public and scientific attention (Zbyryt et al., 2020). Participatory science is also vulnerable to biases through uneven recording or reporting, with greater reporting of individuals or species of special interest (e.g., charismatic or novel species) and uneven spatial coverage of areas that are easily accessible or popular among tourists (Boakes et al., 2016).

Our evidence suggests that observations in the QENP most likely represent a single leucistic individual that was documented many times. Though participatory science can introduce uncertainty about the correlation between observations and individuals (Boakes et al., 2016), observations supplemented with photographs can help distinguish individuals with unique physical characteristics一such as hypopigmented individuals. In our study, all observations were of malachite kingfishers with all white plumage with the exception of an eBird and iNaturalist observation that showed a dark feather on the left wing, which could be explained by either the dark feather being molted over time or not visible in other observations or indicating the possibility of multiple hypopigmented individuals. The disappearance of this dark feather could imply that this individual may be in the late stages of progressive graying rather than affected by true leucism. It would be difficult, however, to confirm or deny either case without confirmed documentation of this individual early in life. Some areas are known to have notable increases of hypopigmentation in populations of other species, reportedly through an inherited recessive mutation (i.e., Asinara donkeys [*Equus africanus asinus var*] in Asinara National Park; Utzeri et al., 2015). However, the relatively short documentation period (1.5 years) in comparison to the average malachite kingfisher lifespan (6.3 years; Rose, 2017) suggests it is unlikely there were multiple leucistic individuals. Therefore, due to the small number and localized incidence of similar observations, we believe it is likely that leucistic observations of malachite kingfishers in our study area represent a single individual.

Participatory science actively engages people from various communities to become involved in scientific research and conservation, while providing researchers with a higher quantity of data than is typically available from traditional research methods (Sullivan et al., 2014). Participatory science records of color aberrations could provide a base to hypothesize on evolutionary timelines (Aguillon & Shultz, 2023). Although participatory science has many positive benefits, such as helping bridge the growing disconnection between people and nature, there are limitations that should be considered when using these data for science. Community involvement in scientific research can sometimes lead to inaccurate data and misidentified observations. For example, studies using iNaturalist data on Seoul frog (*Pelophylax chosenicus*) and Spotless tree toad (*Dryophytes suweonensi*) have shown clear differences in accuracy between experts and novices, with the majority of inaccurate reports based on location (Koo et al., 2022). However, a study of over 104,000 images of 377 passerine species documented in eBird showed that overall, 97% of photographed observations were correctly identified (Gorleri et al., 2022). In addition, as we used photographs of birds to determine pigmentation type, we were able to visually confirm species identifications in our study area. Another key limitation of participatory science is that we often only know if an animal was present, but we do not necessarily know metrics for effort or days spent where nothing was seen. This can make accurately estimating abundance difficult. Having data supplemented with photographs can help alleviate this by highlighting phenotypically distinct characteristics, but it is still an imperfect means of data collection that could be improved in the future to better account for abundance.

Leucism can have negative impacts on an individual's fitness, as color‐aberrant individuals may face increased risk of predation, higher susceptibility to feather weakening, harassment by conspecifics, and difficulty being accepted by a mate (Gayen et al., 2022). One of the eBird observations in our study noted that a mature leucistic kingfisher was seen being fed by a suspected mate with normal coloration (https://macaulaylibrary.org/asset/204207231). We assume the leucistic individual is an adult, because the individual had been documented at least 70 days prior to this observation fully fledged. Courtship among kingfishers includes males feeding females (Davis & Graham, 1991), suggesting that the leucistic individual is likely female and was reproductively active. For this individual, color aberration did not appear to inhibit their ability to find a mate in the wild, suggesting a possible limited effect on their fitness. Additional studies of leucism are needed, however, to determine if disparities are present between sexes.

Color aberration is understudied in kingfishers, we hope our study helps provide a foundation for further research into color aberrations in malachite kingfishers or similarly understudied species. While we only found four previous studies of hypopigmented kingfishers, there were another three documentations that were not peer‐reviewed (Aggarwal, 1991; Gunawardana, 1993; Soni, 1992). We encourage researchers to publish documentation of color aberrations as a way to understand their frequency and ecological consequences. Participatory science provides researchers a starting point for such research by documenting color aberrations in particular species or locations, allowing researchers opportunities to understand trends in occurrence and other patterns. Data from participatory science could prove valuable for studies on other rare ecological occurrences or changes in species distributions (Allen et al., 2022; Williams et al., 2024).

## AUTHOR CONTRIBUTIONS

**Bethany H. Warner:** Conceptualization (supporting); investigation (lead); writing – original draft (lead). **Katherine C. B. Weiss:** Writing – review and editing (equal). **Maximilian L. Allen:** Conceptualization (lead); investigation (supporting); writing – review and editing (equal).

## Supporting information


Data S1.

## Data Availability

The data used in this analysis is are freely available from iNaturalist (www.inaturalist.com) and eBird (https://ebird.org) and is also included as supplementary material.
